# Comprehensive approach to simultaneous molar intrusion and canine retraction in the treatment of Class II anterior open bite using miniscrew anchorage

**DOI:** 10.1590/2177-6709.25.3.30.e1-12.onl

**Published:** 2020

**Authors:** Kaori Shirasaki, Yoshihito Ishihara, Hiroki Komori, Takashi Yamashiro, Hiroshi Kamioka

**Affiliations:** 1 Okayama University, Graduate School of Medicine, Dentistry and Pharmaceutical Sciences, Department of Orthodontics (Okayama, Japan).; 2 Okayama University Hospital, Department of Orthodontics (Okayama, Japan).; 3 Osaka University, Graduate School of Dentistry, Department of Orthodontics and Dentofacial Orthopedics (Suita, Japan).

**Keywords:** Open bite, Orthodontic anchorage procedures, Angle Class II, Tooth movement technique

## Abstract

**Introduction:**

Anterior open bite is one of the most difficult malocclusions to correct in orthodontic treatment. Molar intrusion using miniscrew anchorage has been developed as a new strategy for open bite correction; however, this procedure still has an important concern about prolonged treatment duration in the patient with anteroposterior discrepancy due to the separate step-by-step movement of anterior and posterior teeth.

**Objective:**

This article illustrates a comprehensive orthodontic approach for dentoalveolar open bite correction of an adult patient, by using miniscrew.

**Case report:**

A woman 19 years and 5 months of age had chief complaints of difficulty chewing with the anterior teeth and maxillary incisor protrusion. An open bite of -2.0 mm caused by slight elongation of the maxillary molars was found. The patient was diagnosed with Angle Class II malocclusion with anterior open bite due to the vertical elongation of maxillary molars. After extraction of the maxillary first premolars, concurrent movements of molar intrusion and canine retraction were initiated with the combined use of sectional archwires, elastic chains and miniscrews.

**Results:**

At 4 months after the procedure, positive overbite was achieved subsequent to the intrusion of maxillary molars by 1.5 mm and without undesirable side effects. Class I canine relation was also achieved at the same time. The total active treatment period was 21 months. The resultant occlusion and satisfactory facial profile were maintained after 54 months of retention.

**Conclusion:**

The presented treatment shows the potential to shorten the treatment duration and to contribute to the long-term stability for open bite correction.

## RESUMO

**Introdução:**

A mordida aberta anterior é um dos tipos de má oclusão mais difíceis de se corrigir no tratamento ortodôntico. A intrusão de molares usando ancoragem em mini-implantes foi desenvolvida como uma nova estratégia para a correção da mordida aberta. Entretanto, ainda há preocupações quanto à longa duração desse tratamento em pacientes com discrepâncias anteroposteriores, já que os dentes anteriores são movimentados em etapa distinta dos posteriores.

**Objetivo:**

Este artigo descreve uma abordagem ampla para a correção da mordida aberta dentoalveolar em uma paciente adulta, usando mini-implantes.

**Relato de caso:**

Uma paciente de 19 anos e 5 meses de idade procurou tratamento apresentando como queixas principais a dificuldade de mastigar com os dentes anteriores e a protrusão dos incisivos superiores. Verificou-se, ainda, mordida aberta de -2,0 mm, causada por um comprimento levemente maior dos molares superiores. A paciente foi diagnosticada com má oclusão de Classe II de Angle com mordida aberta anterior devido à dimensão vertical aumentada dos molares superiores. Após a extração dos primeiros pré-molares superiores, iniciou-se movimentos simultâneos de intrusão dos molares e retração dos caninos, com o uso combinado de arcos seccionados, elásticos em cadeia e mini-implantes.

**Resultados:**

Após quatro meses em tratamento, alcançou-se uma sobremordida positiva, devido à intrusão de 1,5 mm dos molares superiores, sem qualquer efeito colateral indesejável. Ao mesmo tempo, também alcançou-se relação de Classe I nos caninos. O tempo total de tratamento ativo foi de 21 meses. A oclusão e o perfil facial satisfatório alcançados ao fim do tratamento mantiveram-se após 54 meses em contenção.

**Conclusão:**

A abordagem aqui apresentada tem o potencial de encurtar a duração do tratamento e contribuir para a estabilidade em longo prazo da correção da mordida aberta.

## INTRODUCTION

Successful correction of anterior open bite is considered one of the most difficult task in orthodontics, and ensuring the long-term stability of the treatment outcome is an important factor to be considered when choosing the method of treatment for patients with anterior open bite.^1^ ^,^ ^2^ Combined surgical-orthodontic treatment is mainly proposed as the common treatment approach for adult patients with severe skeletal maxillomandibular discrepancy.^3^ ^,^ ^4^ Conventional orthodontic treatment methods, such as multiloop edgewise archwire (MEAW)^5^ and nickel-titanium wire with intermaxillary elastics,^6^ have also been used for patients who are reluctant to undergo surgery for open bite correction. Although the patients treated with these modalities achieved adequate overbite, the changes were mainly caused by the extrusion of the anterior teeth, since molar intrusion is relative to incisors extrusion due to the intermaxillary elastics.^7^ Such compensatory eruption of incisors is often undesirable for patients who also have maxillary vertical excess, a long face, or excessive gingival display.

In recent years, several studies have demonstrated effective molar intrusion in the treatment of anterior open bite patients using temporary anchorage devices (TADs).^8^ ^-^ ^10^ This new treatment strategy resulted in counterclockwise rotation of the mandible, a reduction in the anterior vertical facial height, advancement of the chin, and improvement of the retrognathic appearance of the facial profile without surgical intervention or incisal elongation. However, the overall treatment duration would be prolonged in cases of anterior open bite with anteroposterior discrepancy, as the anterior teeth would be moved after intrusion of the molars using other methods.

In this article, an approach of treatment of an adult patient with dentoalveolar open bite using miniscrews is presented. An efficient method of simultaneous molar intrusion and canine retraction is reported to shorten the treatment duration for correcting open bite with minimal side effects. In addition, information about the long-term stability throughout 4.5 years of retention is provided.

## DIAGNOSIS AND ETIOLOGY

**Figure 1:**
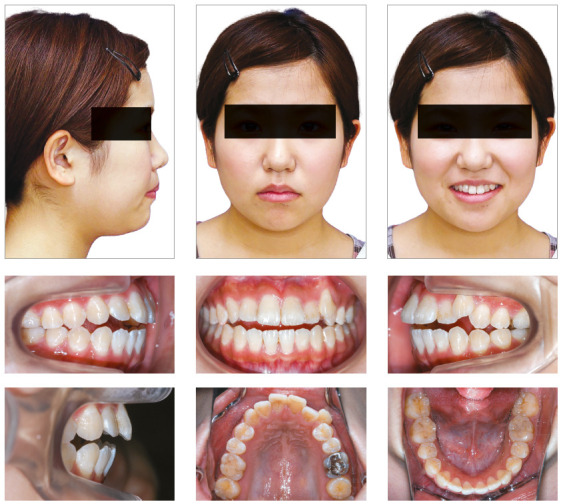
Pretreatment facial and intraoral photographs.

**Figure 2:**
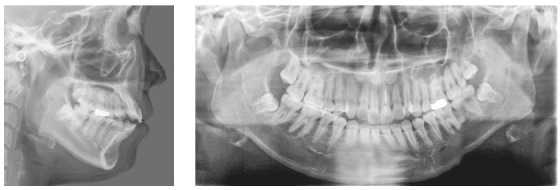
Pretreatment lateral cephalogram and panoramic radiograph.

A woman 19 years and 5 months of age came to the outpatient clinic of Okayama University Hospital. Her chief complaints were difficulty chewing with the anterior teeth and maxillary incisor protrusion. She had a convex profile and suffered from circumoral musculature strain to achieve lip seal (Fig 1). A decreased overbite of -2.0 mm with Angle Class II molar relationships on both sides was observed. In addition, she had two distinct occlusal planes within the maxilla. Anterior shift of the mandible could be found due to occlusal interference positively associated with the crossbite on the left first premolars. Mild crowding was also observed in the maxillary incisors. The maxillary dental midline was shifted 0.5 mm toward the left of the facial midline. The mandibular dental midline almost coincided with the facial midline (Fig 1). A dental panoramic radiograph revealed asymmetry of both condyles and mandibular ramus without pathological problems in the root structure or the periodontal condition (Fig 2). The patient reported clicking sounds in the temporomandibular joint on the left side. The interincisal distance on maximum opening without pain was 43 mm. Her gingival display on smiling was acceptable.

Compared with Japanese norms,^11^ a cephalometric analysis showed a skeletal Class I jaw relationship (ANB = 4.0°) with a low mandibular plane angle (Mp-FH, 24.0°), a small gonial angle (111.0°), and a normal anterior facial height (N-Me = 126.0 mm). The maxillary incisors were slightly inclined labially (U1-FH = 117.0°), and the mandibular incisors were significantly inclined labially (L1-Mp = 110.0°). The maxillary molars were slightly extruded (U6/PP = 27.0 mm) (Table 1).

**Table 1:** Summary of cephalometric findings.

Variable	Japanese norms for women	SD	pretreatment	post-treatment	2 years post-retention	4.5 years post-retention
Angular (degrees)						
ANB	2.8	2.44	4.0	5.0	5.0	5.0
SNA	80.8	3.61	82.0	82.0	82.0	82.0
SNB	77.9	4.54	78.0	77.0	77.0	77.0
FMA	30.5	3.6	24.0	24.0	24.0	24.0
Go.A	122.1	5.29	111.0	111.0	111.0	111.0
U1-FH	112.3	8.26	117.0	104.5	104.5	104.5
L1-Mp	93.4	6.77	110.0	100.0	102.0	102.0
interincisal angle	123.6	10.64	109.0	131.5	129.5	129.5
Linear (mm)						
S-N	67.9	3.65	64.5	64.5	64.5	64.5
N-Me	125.8	5.04	126.0	126.0	126.0	126.0
Me/PP	68.6	3.71	70.0	70.0	70.0	70.0
Ar-Go	47.3	3.33	55.0	55.0	55.0	55.0
Go-Me	71.4	4.14	72.0	72.0	72.0	72.0
Ar-Me	106.6	5.74	109.0	109.0	109.0	109.0
Overjet	3.1	1.07	4.0	2.5	2.5	2.5
Overbite	3.3	1.89	-2.0	2.5	2.5	2.5
U1/PP	31	2.34	30.0	30.5	30.5	30.5
U6/PP	24.6	2	27.0	25.5	25.5	25.5
L1/Mp	44.2	2.68	43.0	43.5	43.5	43.5
L6/Mp	32.9	2.5	35.0	36.5	36.5	36.5

## TREATMENT OBJECTIVES

The patient was diagnosed with skeletal Class I jaw-base relationship, an Angle Class II malocclusion with a low mandibular plane angle, and an anterior dentoalveolar open bite. The treatment objectives were to correct the anterior open bite and establish ideal overjet and overbite, to achieve an acceptable occlusion with a good functional Class I occlusion, and to improve her facial profile. In this case, the maxillary first premolars and mandibular second premolars were extracted to achieve the treatment objectives. The use of miniscrews to achieve intrusion of the elongated maxillary molars to correct the dentoalveolar open bite was planned.

## TREATMENT ALTERNATIVES

The first treatment option involved comprehensive orthodontic-surgical treatment. The patient considered this approach too aggressive and invasive. In addition, she wished to avoid orthognathic surgery. 

Another proposed treatment option involved a conventional orthodontic treatment, such as the MEAW technique, without skeletal anchorage. This option would have led to slight extrusion of both the incisors, as described above; nonetheless, the vertical relationship of the patient’s incisors and upper lip was considered acceptable before orthodontic treatment. 

Miniscrews have a lower surgical burden and cause less discomfort to the patient than traditional procedures. This patient had two distinct occlusal planes, and the extruded maxillary molars were suggested as a main cause of the dentoalveolar open bite. Therefore, intrusion of the extruded maxillary molars was considered to be the a more appropriate treatment choice. This would have led to the autorotation of the mandible in the counterclockwise direction, thereby increasing the overbite and improving the convex profile. After discussing each treatment options, the patient selected the miniscrew-assisted treatment.

## TREATMENT PROGRESS

**Figure 3:**
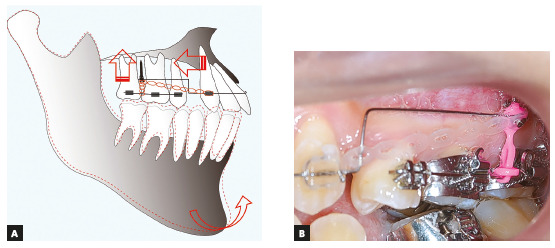
A) Schematic illustration of simultaneous molar intrusion and canine retraction procedure. (B) Miniscrew implanted at the distal alveolus of the maxillary first molars.

**Figure 4:**
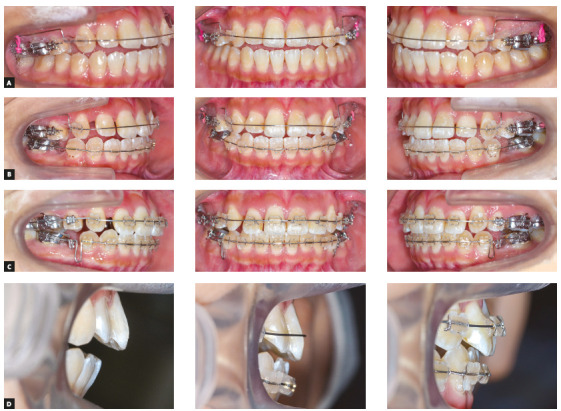
The treatment progress during simultaneous molar intrusion and canine retraction. (A) At the start of simultaneous molar intrusion and canine retraction. (B) Two months after the start of simultaneous molar intrusion and canine retraction. (C) Four months after the start of simultaneous molar intrusion and canine retraction. (D) Representative intraoral photographs showing gradual changes in the incisor relationships.

Following the extraction of the maxillary first premolars, two titanium miniscrews (1.6 mm diameter; 6 mm length; Absoanchor^®^; Dentos Ltd., Daegu, South Korea) were implanted at the distal alveolus of the maxillary first molars (Fig 3). A transpalatal arch appliance was also placed between the maxillary first molars to compensate for the crown buccal torque that would be caused by the intrusion force. A 0.018-in preadjusted Edgewise appliance was placed on the maxillary molars. After leveling and alignment with nickel-titanium archwires for two months, 0.016×0.022-in stainless-steel archwires were installed to initiate an orthodontic intrusive load of 100g on the maxillary molars (Fig 4A). Simultaneously, 0.018-in preadjusted Edgewise appliances were also placed into the maxillary canines. A 0.016-in stainless-steel archwire was installed and ligated to the miniscrews to induce space closure of the maxillary arch using sliding mechanics (Fig 4B, C). This method for achieving simultaneous molar intrusion and canine retraction enabled the achievement of both a positive overbite and a maxillary canine retraction during four months of treatment without undesirable side effects (Fig 4D). The maxillary occlusal plane was also flattened.

Extraction of the mandibular second premolars resulted in a more retruded position of the mandible. This shift was probably due to the removal of occlusal interference. Class II intermaxillary elastics were used for three months to assist the mesial movement of the mandibular molars. 

After removing the appliances and the miniscrews, the maxillary and mandibular teeth were stabilized using a 6-unit lingual bonded retainer and a wraparound-type retainer. The total active treatment period was 21 months.

## RESULTS

**Figure 5:**
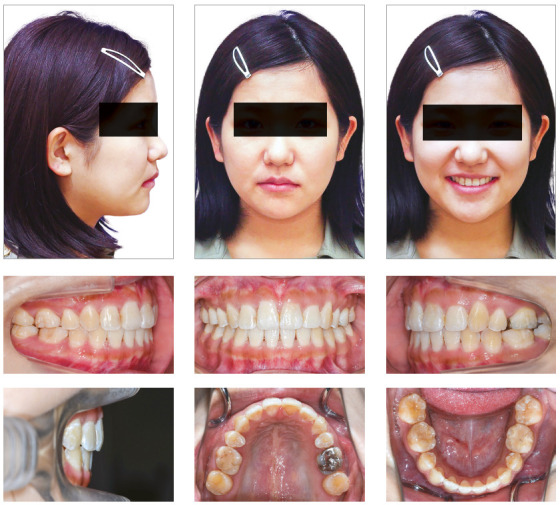
Posttreatment facial and intraoral photographs.

**Figure 6:**
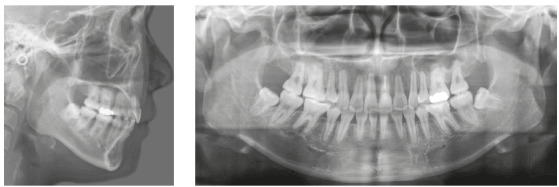
Posttreatment lateral cephalogram and panoramic radiograph.

**Figure 7:**
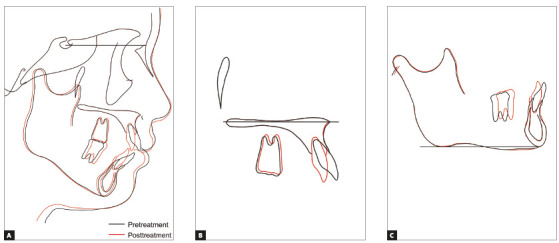
Superimposed cephalometric tracings showing changes from pretreatment to posttreatment: A) Sella-nasion plane at sella, B) Palatal plane at ANS, C) Mandibular plane at menton.

**Figure 8:**
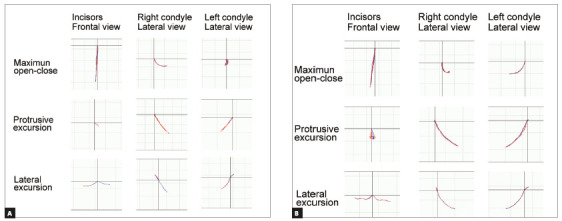
Condylar movement and incisal paths recorded with the six degrees of freedom jaw movement recording system. The red lines indicate the opening phase, and the blue lines indicate the closing phase: A) Pretreatment, B) Posttreatment.

**Figure 9:**
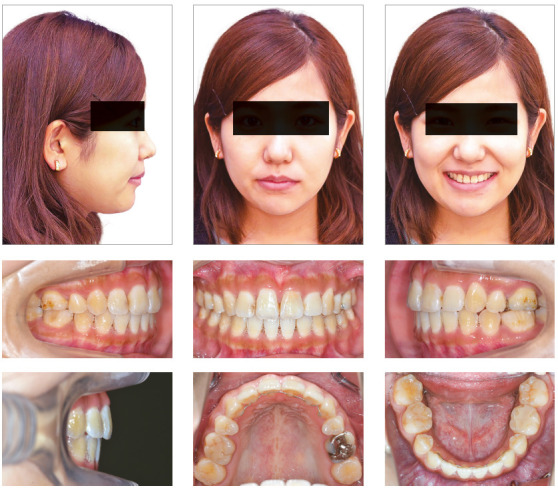
Two-year retention facial and intraoral photographs.

**Figure 10:**
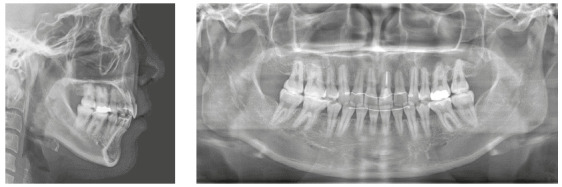
Two-year retention lateral cephalogram and panoramic radiograph.

**Figure 11:**
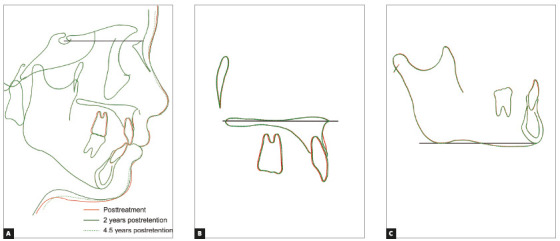
Superimposed cephalometric tracings showing changes from posttreatment to 4.5 years of retention: A) Sella-nasion plane at sella, B) Palatal plane at ANS, (C) Mandibular plane at menton.

**Figure 12:**
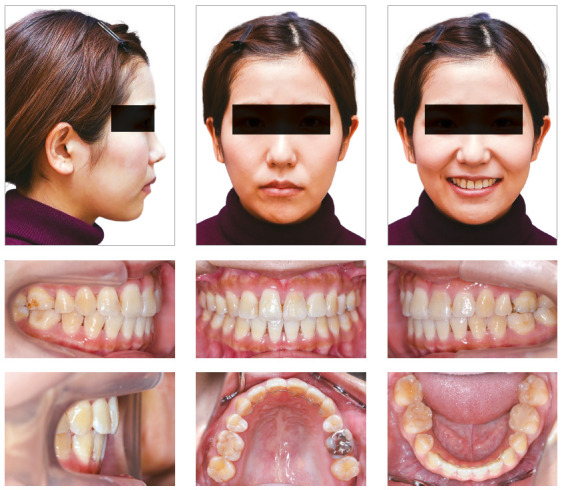
Four-and-a-half-year retention facial and intraoral photographs.

The posttreatment facial photographs showed a balanced and harmonious facial profile due to the upper and lower lips retraction, reducing the strain of the mentalis muscle on lip closure. The dental midlines were coincident with the facial midline (Fig 5). A Class I molar relationship and an acceptable interincisal relationship were established on both sides. The posttreatment intraoral photographs also showed well-aligned arches, a flattened occlusal plane, and the good interdigitation of the teeth (Fig 5). Acceptable root parallelism was observed on the panoramic radiograph. Slight apical root resorption was observed, especially in the maxillary incisors (Fig 6). A posttreatment cephalometric evaluation and the superimposed cephalometric tracing showed no marked skeletal changes. The maxillary first molars were intruded 1.5 mm toward the palatal plane, whereas both the maxillary and the mandibular incisors were extruded by 0.5 mm (Fig 7). In an evaluation of the jaw movement with a jaw movement recording system with six degrees of freedom (Gnathohexagraph system, v. 1.31; Ono Sokki, Kanagawa, Japan),^12^ a smooth and stable incisal path was found during protrusive or lateral excursion. Furthermore, an increase in the condylar movement was also observed on the left side during maximum opening mandibular movement (Fig 8). The interincisal distance on maximum opening without pain increased to 50 mm. After a 2-year retention period, the patient’s occlusion was stable, and the favorable facial profile achieved by the orthodontic treatment was also maintained (Fig 9). Post-retention intraoral photographs also showed that the molar and canine relationships, respectively, had been maintained whereas slight reopening of the extraction space between right second premolar and first molar (Fig 9). A cephalometric analysis over the 2-years post-retention period showed a slight forward movement of the maxillary dental arch (Fig 10). In addition, the mandibular incisors were labially inclined by 2° (Fig 11, Table 1). There was no significant difference between the retention at 2 years and that at 4.5 years (Fig 12, Table 1). The acceptable occlusion and ideal overbite and overjet were well maintained, and the patient was satisfied with the treatment results.

## DISCUSSION

The orthodontic correction of the anterior open bite can be achieved by several mechanisms that will result in the intrusion of posterior teeth, the extrusion of anterior teeth, or a combination of both. When the treatment of the dentoalveolar open bite is considered, leveling a maxillary arch by inserting a continuous archwire promotes extrusion of the incisors that is sometimes undesirable in adult patients. This can be explained by the leveling of a maxillary arch with two distinct occlusal planes, which is a common characteristic of a dentoalveolar open bite.^13^ The principle of segmental molar intrusion involves minimizing any adverse side effect before leveling and retracting the anterior teeth. However, these separate step-by-step movements of the anterior and posterior teeth can prolong the overall treatment duration.

In the present report, we demonstrated a concurrent approach for managing molar intrusion and canine retraction with miniscrew-aided mechanics. The method, which involved a combination of three segmented archwires, miniscrews, and a transpalatal arch, enabled us to control the anterior and posterior segments independently. This approach is clinically relevant because the vertical and anteroposterior problems can be addressed simultaneously, resulting in a shorter treatment duration. Our simple design proposed in this study is easy to fabricate, requires no patient cooperation, is less likely to cause soft tissue impingement, and allows clinicians to deliver well-controlled orthodontic force with minimum chair-side adjustment. In addition, a shorter treatment duration may also desirable from the perspective of exposure to possible undesirable side effects of orthodontic treatment, especially the shortening of tooth roots.

The occurrence of apical root resorption is an undesirable but frequent side effect for patients who undergo orthodontic treatment.^14^ Lund et al.^15^ reported that practically all patients and up to 91% of all teeth showed some degree of root shortening. Some of the risk factors of root resorption are very controversial, but previous reports have concluded that open bite^16^ ^,^ ^17^ and a prolonged treatment duration^18^ ^,^ ^19^ are high-risk factors for apical root resorption. In the present case, no force was applied to move the maxillary incisors in the early stage of the treatment, so there were no potentially detrimental effects on the teeth, such as round-trip movement. Consequently, we think that our system might help reduce the risk of severe root resorption in the upper incisors due to the small amount of time for which Edgewise appliances were mounted in this patient.

Pretreatment records revealed asymmetric mandibular morphology and condylar movement (Fig 8). Although determining the cause of an anterior open bite is complicated, this asymmetry may be related to the functional impairment-induced resorption of the condyles and consequent shortening of the mandibular rami, leading to the development of a Class II anterior open bite.

We used Class II intermaxillary elastics to move the mandibular molars mesially in the later stage of the treatment. Although the patient’s mandible did not rotate toward the clockwise direction, this procedure led to the slight extrusion of the mandibular molars. Several reports have described no mandibular advancement or correction of the convex profile by molar intrusion in a single jaw due to the adverse extrusion of molars in the opposite jaw.^20,21^ In the present patient, it was not necessary to reduce the facial height because she already had an aesthetically balanced facial height at pretreatment.

Many orthodontists remain concerned about the long-term stability of molar intrusion using TADs. A high prevalence of relapse of molars has been reported within the first^22^ ^,^ ^23^ or second years of retention.^20^ Several studies have also examined various risk factors influencing the relapse of open bite correction, such as a long divergent skeletal pattern^24^ and non-extraction treatment.^25^ Our patient showed acceptable retention up to 4.5 years post-retention, with a low mandibular plane angle and the extraction of four premolars. We believe that our treatment choices contributed to the good stability of the occlusion. Although limited information is available and further studies will be needed to determine the prognosis, these findings provide information to help achieve further favorable stability in this patient.

## CONCLUSION

This report described comprehensive biomechanics for achieving simultaneous molar intrusion and canine retraction in the treatment of anterior open bite using miniscrew anchorage. This treatment method enabled the independent control of both the anteroposterior and vertical dimensions with optimum force and minimum side effects. The resultant occlusion and facial profile were stable after 4.5 years. These results suggest that our system is an effective approach in the treatment of dentoalveolar open bite patients with anteroposterior discrepancy for attaining functionally stable occlusion with a short treatment duration.
